# Social Media as a Platform for Recruitment to a National Survey During the COVID-19 Pandemic: Feasibility and Cost Analysis

**DOI:** 10.2196/28656

**Published:** 2021-07-06

**Authors:** Heidi Green, Ritin Fernandez, Catherine MacPhail

**Affiliations:** 1 School of Nursing University of Wollongong Wollongong Australia; 2 Centre for Research in Nursing and Health St George Hospital Kogarah Australia; 3 School of Health and Society University of Wollongong Wollongong Australia

**Keywords:** social media, survey, online recruitment, COVID-19, pandemic, methodology

## Abstract

**Background:**

With improved accessibility to social media globally, health researchers are capitalizing on social media platforms to recruit participants for research studies. This has particularly been the case during the COVID-19 pandemic, when researchers were not able to use traditional methods of recruitment. Nevertheless, there is limited evidence on the feasibility of social media for recruiting a national sample.

**Objective:**

This paper describes the use of social media as a tool for recruiting a national sample of adults to a web-based survey during the COVID-19 pandemic.

**Methods:**

Between August and October 2020, participants were recruited through Facebook via two advertisement campaigns (paid option and no-cost option) into a web-based survey exploring the relationship between social determinants of health and well-being of adults during the COVID-19 pandemic. Data were analyzed using SPSS software and Facebook metrics that were autogenerated by Facebook Ads Manager. Poststratification weights were calculated to match the Australian population on the basis of gender, age, and state or territory based on the 2016 Australian census data.

**Results:**

In total, 9594 people were reached nationally with the paid option and potentially 902,000 people were reached through the no-cost option, resulting in a total of 1211 survey responses. The total cost of the advertisement campaign was Aus $649.66 (US $489.23), resulting in an overall cost per click of Aus $0.25 (US $0.19).

**Conclusions:**

Facebook is a feasible and cost-effective method of recruiting participants for a web-based survey, enabling recruitment of population groups that are considered hard to reach or marginalized. Recruitment through Facebook facilitated diversity, with participants varying in socioeconomic status, geographical location, educational attainment, and age.

## Introduction

Numerous strategies such as newspaper advertisements, random mail out of surveys, and random digit dialing have been used to recruit participants into population health research. However, implementation of these traditional strategies in modern society has limitations due to the reduced use of landline phones and increased postage costs [1,2], which make these recruitment methods less feasible. Additionally, these approaches have low participation rates ranging from 7.5% [3] to 30% [4]. With improved access to the internet globally, particularly through mobile phones, social media has become an active part of modern society [5]. Public health researchers have harnessed social media and web platforms as a modality for recruitment into population health research [6,7]. Used as more than just a method to connect with friends and family, social media platforms are increasingly used for sharing content, engaging with news content, entertainment, and receiving health information. The most popular social media platforms globally are Facebook, Twitter, YouTube, and Instagram [8], with over 4 billion users. Social media platforms enable users to connect and share information through both traditional and interactive methods, with most platforms allowing free use [9].

According to the Australian Communications and Media Authority [10], in 2018-19, approximately 91% of all Australians had access to the internet. In 2016-17, 80% of Australians used the internet for social networking [11] compared with 66% in 2011 [12], with an average of 1.2 social media accounts per Australian [8]. Facebook is the most popular social media platform among Australians, with approximately 93% of Australian social media consumers using this platform, followed closely by Instagram at 73% [13]. Moreover, almost 60% of Australians use social media daily [8].

Given the increased prevalence of daily social media use among Australians, social media platforms have been increasingly used as a viable method for recruiting participants into health research [14]. More specifically, social media platforms allow researchers to access hard-to-reach populations as well as target recruitment through the use of advertising campaigns to specific users based on gender, geographical location, interests, and age [9]. Social media use has been harnessed by heath researchers to recruit participants into a range of studies, including cross-sectional studies, observational studies, and interventional studies [5], particularly due to the cost-effectiveness of this recruitment method. There is evidence in the literature that health researchers have recruited participants and delivered health behavior interventions on a variety of topics. The success of these interventions has demonstrated the efficacy of social media as a suitable method for accessing participants [1,5,15-17]. However, a substantial number of studies use a localized sample.

Our study engaged the use of social media with the purpose of generating a national sample of Australian adults to explore the relationship between the social determinants of health and well-being during the COVID-19 pandemic. Currently, there is limited evidence available on the feasibility of social media for recruiting a national sample. Therefore, the aim of this paper is to describe the feasibility of using social media as a tool for recruiting a national sample of adults to a web-based survey during the COVID-19 pandemic. Feasibility was assessed in terms of reach, time invested in recruitment, number of surveys completed, cost-effectiveness, and recruitment of a diverse sample of participants.

## Methods

### Study Overview

The research study was undertaken to investigate the relationship between social determinants of health and well-being in Australian adults during the COVID-19 pandemic. Ethical approval to conduct this study was received from University of Wollongong Human Ethics Committee (2020/306). The inclusion criteria for the study were individuals aged 18 years and above, with the ability to read English and residing in any state or territory within Australia. Participants were recruited using Facebook over a 9-week period between August and October 2020. Participants were required to complete a web-based survey comprising 49 questions exploring social determinants of health. They were invited to enter a draw to win one of 10 Aus $50 gift vouchers at the end of the survey with winners selected randomly using SPSS software (version 25). A currency exchange rate of Aus $1=US $0.75 is applicable.

### Recruitment Strategy

Recruitment for this study using Facebook was achieved by the following two methods: (1) joining existing community noticeboard Facebook groups (ie, no-cost option), and (2) through a paid Facebook advertisement campaign (ie, paid option). Both methods enabled snowball sampling where users could like, share, and circulate the social media post among others.

#### Joining Existing Community Noticeboard Groups on Facebook (No-Cost Option)

A specific Facebook page was created for the study using a study image. To ensure national representation, the primary author (HG) identified existing Facebook community noticeboard groups, according to Australian states and territories as well as based on urban, regional, and remote areas. The author contacted the administrators of each individual community group for permission to join. Each week, if permitted by the administrators, the advertisement was reposted on each of the community noticeboard group pages. Posting on the existing community noticeboard groups began on August 20, 2020, and ended on October 14, 2020.

#### Facebook Advertising Campaign (Paid Option)

To supplement the no-cost Facebook community noticeboard group approach, a paid advertisement through Facebook, which included Instagram, was designed to recruit participants. Two consecutive advertisement campaigns were set up, with the first campaign used to establish the feasibility of this strategy.

The Facebook advertisement platform, Facebook Ads Manager, was used to create paid advertisements. The features available for a payment allows the advertisement to be customized based on objective (eg, links or clicks to a web-based survey), target audience (eg, location, age, gender, interests, and behaviors), budget, and schedule [18]. Selecting the “automatic placements” option when setting up the advertisement in Facebook Ads Manager allowed the advertisements to run across associated services such as Instagram, Messenger, and Facebook Audience Network (ie, off-Facebook in-app advertising network for mobile apps).

**Figure 1:**
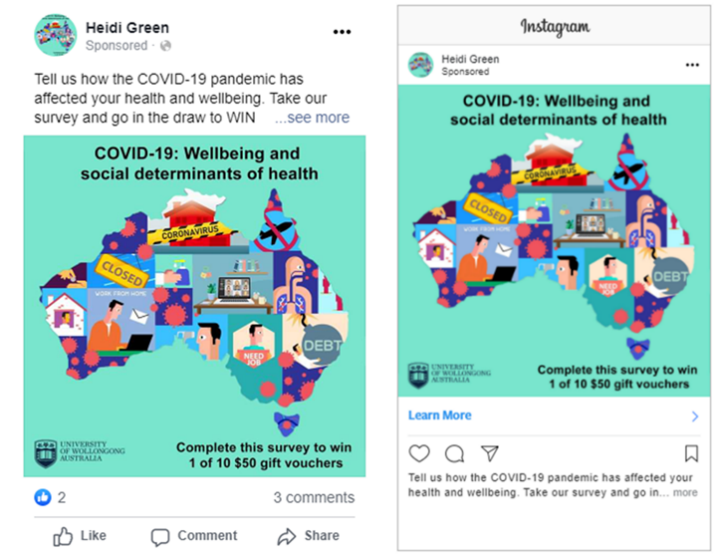
Paid Facebook and Instagram advertisements—example post.

These Facebook advertisements comprised a main text (eg, “Tell us how the COVID-19 pandemic has affected your health and wellbeing. Take our survey and go in the draw to WIN 1 of 10 Aus $50 gift vouchers”), an image (ie, the study image and university logo), and display link (Figure 1).

A budget of Aus $650 was set as the maximum recruitment spend for the paid campaigns, with a daily limit of Aus $25. The cost per click can vary depending upon the number of clicks on the advertisement and the amount of the daily budget reached.

The first campaign was set as “engagement” (targeting people most likely to engage with the post through one of the following mechanisms: share, like, or click). The target audience for the first campaign was (1) people residing in Australia, (2) people aged 18-35 years inclusive, (3) people of all genders, and (4) people residing within certain postcodes. The primary researcher used the Australian Bureau of Statistics (ABS) Index of Relative Socio-Economic Advantage and Disadvantage (IRSAD) to set these specific postcodes. These postcodes were used to ensure the distribution of the ad campaign targeted potential participants in both relative advantaged and disadvantaged locations. The “automatic placements” option on Facebook was used, which allows the campaign to maximize the set budget and dissemination of the advertisement to a larger sample relevant to the inclusion criteria [18].

Next, the “post engagement” strategy was selected, enabling delivery to the people who are likely to share, like, and comment on the post at the lowest cost [18]. The first Facebook advertisement campaign ran from August 25, 2020, to September 1, 2020.

The second campaign employed the same strategies as the first advertisement campaign; however, the target audience locations were identified using suburbs set by ABS’s IRSAD. This was undertaken as suburbs can contain multiple postcodes thus increasing the target audience. The use of the ABS’s IRSAD suburbs allowed a general representation of both advantaged and disadvantaged locations, enabling diversity in targeting potential participants. The second campaign ran from September 6, 2020, to September 22, 2020.

Throughout the recruitment period, the Facebook posts were monitored daily to ensure that any comments, including individuals opportunistically using the advertisement to promote businesses, were hidden from other Facebook users. This was undertaken to ensure potential respondents were not influenced to either participate or be discouraged from participating in the survey. Additionally, monitoring the comments and hiding them from other potential participants was conducted for ethical reasons as a way of protecting any potential participants’ identities. Automatic hiding of comments is not available as an option within Facebook’s delivery system and, therefore, it had to be conducted manually.

### Data Analysis

Data were analyzed using SPSS software (version 25). Poststratification weights were calculated to match the Australian population on the basis of gender, age, and state or territory based on the 2016 Australian census [19], to account for over- or underrepresentation of certain people.

Facebook metrics were collected through Facebook Ads Manager, which auto generates the engagement activity for each advertisement campaign [18]. Summary and descriptive statistics including reach, impressions, and cost per click were analyzed for each campaign and for the overall campaign. “Reached” refers to the number of people who were shown the advertisement, “impressions” refers to the number of times the advertisement was on-screen for the target audience and could include multiple views of the advertisement by the same individual. “Cost per click” is derived from the total advertisement campaign spend divided by the number of clicks on the advertisement or the link [18].

## Results

### Recruitment Through Facebook (No-Cost Option)

The primary researcher (HG) made a request to the administrators of 110 existing Facebook community noticeboard groups to join those groups. All community groups approached approved the author’s request to join. Posts and reposts to the existing community noticeboard group Facebook pages were conducted 10 times over the 9-week period commencing on August 21, 2020, and the last repost made on October 14, 2020. Using this option implies that no data on the individuals reached or impressions recorded is available to researchers through Facebook Ads Manager; however, the number of members in each community noticeboard group were available with a potential reach of 902,000 individuals. Nationally, each community noticeboard group had an average of 8205 group members, with slightly higher than the national average seen for Queensland and Australian Capital Territory, at 11,097 and 12,230 average total members per noticeboard community group, respectively. In contrast, South Australia and Victoria had marginally lower average members per group than the national average, with 6480 and 6287 members, respectively. Additionally, a comparison between the no-cost and paid options to determine the most cost-effective option was not possible, as both recruitment methods sent participants to the same survey link; therefore, no there was disaggregation between the options the participants used to reach the survey page.

### Recruitment Through Facebook (Paid Option)

An aggregated 9594 individuals were reached via the two paid advertisement campaigns; however, a total of 14,232 impressions were recorded. The Facebook advertisement campaign reached 5316 (55.4%) male, 4062 (42.3%) female, and 216 (2.3%) users with uncategorized gender. Using the automatic placements option, most placements were conducted through Instagram, reaching 5846 individuals, whereas Facebook reached 3856 individuals. The remainder of individuals were reached through Facebook Audience Network.

### Strengths and Limitations of Facebook (No-Cost Option)

The greatest advantage in using the no-cost option is that there are no monetary costs associated with recruiting participants. However, it must be noted that the researchers had to continually repost the ad to the community noticeboard groups to ensure visibility, as the post would move down a user’s feed once posts had been posted by another group or member; this in turn proved to be labor intensive. Additionally, during the first few days of recruitment, responses from the no-cost option were received predominantly from individuals aged 35 years and above. Therefore, to supplement this approach, the paid option was used and intentionally designed to target younger potential respondents.

### Strengths and Limitations of Facebook (Paid Option)

The paid option allowed the researchers to specifically target younger potential respondents across not only Facebook but also Instagram, Messenger, and Facebook Audience Network. Furthermore, the paid option allows the researcher to customize the ad based on their objective and to create a specific schedule of when the ads will be seen [18]. This was particularly important to recruit a diverse national sample of participants. The drawback with using the paid option was the associated monetary costs, albeit being able to design the campaign to have a daily limit, the reach of potential participants did not guarantee actual respondents.

### Overall Response to Survey

A total of 1211 individuals responded to the survey, with 100% meeting the eligibility criteria. The survey took respondents approximately 9 minutes to complete. Of the 1211 who commenced the survey, 1137 (93.89%) respondents completed it.

**Figure 2:**
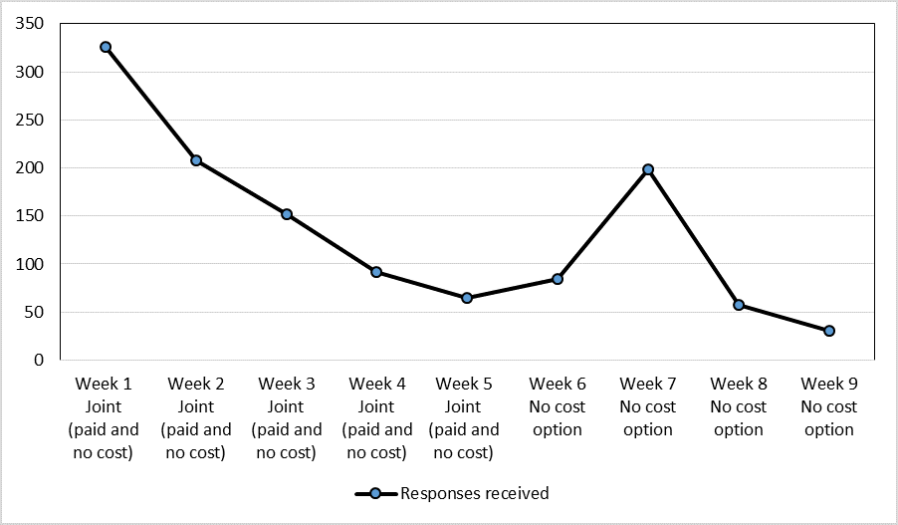
Overall response to the survey (no-cost and paid options).

The number of responses received varied per day among the paid and no-cost recruitment options, with the highest number of responses (n=178) received on August 21, 2020, and the lowest (n=0), on October 21, 2020. In the first week the survey was live, a total of 326 responses were received, which was the most responses received over the 9-week period. Due to the no-cost and paid options running concurrently for the first 5 weeks, using the same survey link, the numbers of participants recruited through each option are unknown. Overall response to the survey per week for the no-cost and paid options are outlined in Figure 2.

### Cost Analysis

For the paid option, the total amount spent on the Facebook advertisement campaigns was Aus $649.66, with the average overall cost per click (per post engagement) reported at Aus $0.25. Individuals aged 18-24 years accounted for Aus $419.79 (64.6%) of the total advertisement budget, whereas individuals in the 25-34 age group accounted for Aus $192.49 (37.1%), those aged 35 years accounted for Aus $37.38 (7.6%). The majority of the advertisement spend was using Instagram, with a total spend of Aus $598.39. Facebook advertisement total spend was Aus $50.79, whereas Aus $0.48 of the total spend was through Facebook Audience Network. The lowest cost per click day was on the 8 September 2020 at Aus $0.16, with the highest cost per click of Aus $0.32 on September 18, 2020.

More male participants engaged with the Facebook advertisement campaign compared to female participants, with the former accounting for 60.4% (Aus $392.35) of the total spend. Women in the 25-34 age group account for the highest cost per click at Aus $0.28.

### Time

Economically, Facebook advertising campaigns are a feasible method to recruit participants into a web-based survey, requiring the use of a single researcher to create, manage, and maintain the recruitment strategy. The total number of hours spent by the researcher, including management of the no-cost option of posting on existing community noticeboard groups within Facebook, was a total of 30 hours over the 9-week period. The benefit of using Facebook’s features of selecting a target audience, and posting on existing community noticeboard groups enabled recruitment of a large sample within a short timeframe, with a relatively low cost of Aus $649.66. The cost-effectiveness and ability to recruit a large sample provides evidence to suggest that Facebook recruitment is a feasible option for public health researchers.

### Distribution of Respondents

Participants from diverse geographic, education, and employment backgrounds were recruited through these two Facebook methods. Responses were received from all states (n=6) and territories (n=2) within Australia. Based on weighted data from 1211 participants, most responses received from New South Wales at 34.4% (n=387), whereas 0.4% (n=5) were received from the Northern Territory. Responses were received from 40.4% (n=447) participants living in locations classified as having the two lowest socioeconomic status brackets and 41.2% (n=646) participants living in locations classified as having two highest socioeconomic status brackets. Responses were received from 662 (58.8%) residents in major cities, 373 (23.1%) residents in inner or outer regional areas, and 70 (6.2%) residents in remote or very remote areas of Australia. Educational attainment varied among the respondents, with 36.1% (n=406) having at least a bachelor’s degree, 20.2% (n=239) having a completed technical college, and 22.2% (n=250) had completed years 7 to 12 of high school. Responses received from those aged 25-39 years and 40-59 years was 30.2% (n=340) and 35.5% (n=40), respectively. The mean age of the respondents was 46.3 (SD 16.3) years. Responses received from female participants accounted for 51.7% (n=582) and that from male participants accounted for 48.3% (n=545). Unweighted data for transgender or nonbinary population was 2.6% (n=30). Weighted and unweighted distribution of respondents are detailed in Table 1.

**Table 1:** Distribution of respondents (nonweighted and weighted).

Characteristic		Nonweighted data	Weighted data
Age (years), mean (SD)		43 (14.2)	46.3 (16.3)
**Age range (years), n (%)**			
	18-24	118 (9.7)	101 (8.9)
	25-40	413 (34.1)	340 (30.2)
	41-60	464 (38.3)	400 (35.5)
	61-75	135 (11.1)	227 (20.2)
	>75	7 (0.6)	59 (5.2)
**Gender, n (%)**			
	Women	938 (80.7)	582 (51.7)
	Men	194 (16.7)	545 (48.3)
	Nonbinary or transgender	30 (2.6)	N/A^a^
**Education, n (%)**			
	Completed years 7 to 12 high school	240 (20.7)	250 (22.2)
	Vocational	253 (21.8)	239 (21.2)
	Bachelor’s degree	437 (37.7)	406 (36.1)
	Postgraduate degree	230 (19.8)	230 (20.4)
**State or territory, n (%)**			
	New South Wales	695 (59.8)	387 (34.4)
	Victoria	181 (15.6)	305 (27.0)
	Queensland	127 (10.9)	219 (19.4)
	Western Australia	91 (7.8)	118 (10.5)
	South Australia	17 (1.5)	57 (5.1)
	Northern Territory	19 (1.6)	5 (0.4)
	Australian Capital Territory	19 (1.6)	18 (1.6)
	Tasmania	13 (1.1)	19 (1.7)
**Remoteness, n (%)**			
	Major cities	709 (62.1)	662 (58.8)
	Inner regional	256 (22.4)	224 (19.9)
	Outer regional	112 (9.8)	149 (13.2)
	Remote	20 (1.8)	12 (1.1)
	Very remote	45 (3.9)	58 (5.1)
**Socioeconomic status, n (%)**			
	Lowest (most disadvantaged)	157 (13.8)	188 (16.6)
	Low	252 (22.1)	259 (23)
	Middle	210 (18.4)	194 (17.2)
	High	193 (16.9)	182 (16.1)
	Highest (most advantaged)	328 (28.8)	282 (25.1)

^a^N/A: not applicable.

## Discussion

### Principal Findings

This study reports on the feasibility of using Facebook to recruit a national sample of participants. The findings demonstrate Facebook to be an efficient and effective method to recruit both a large and diverse sample of respondents. We recruited a total of 1211 respondents, with weighted data demonstrating recruitment was representative of the Australian population. The average cost per click for the paid option was Aus $0.25 with 9594 people reached. The no-cost option potentially reached 902,000 people, with an average number of 8205 members in each community noticeboard group. The findings of this study have implications for public health researchers seeking to recruit study participants through social media sites such as Facebook and contribute to the emerging evidence regarding the ability of social media to reach diverse populations groups.

Overall, the no-cost and paid Facebook advertisements used in this study proved to be an effective method for recruiting a large national sample of the Australian population. Although concerns have been raised in the literature regarding the digital divide [20], the accessibility of Facebook and Instagram globally and nationally refutes this notion [8]. The literature confirms that social media advertisement is a viable method to recruit marginalized population groups and those considered hard to reach [21,22]. The focus of this recruitment strategy was a diverse national sample of adults. The targeted paid advertisements for this study were achieved using the ABS’s IRSAD postcode and suburbs to target a diverse audience, which proved effective, with respondents varying in socioeconomic status, remoteness, educational attainment and age. The representation of regional and remote area–based participants shows the potential benefit of using social media to recruit a segment that traditionally has been quite difficult to reach [14]; this can also be said from those from low-socioeconomic backgrounds [17]. However, it must be noted that gender was not diverse in this study with participants identifying as female overrepresented. This similar to the experience of other studies, in which male, nonbinary, and transgender participants are underrepresented [23,24]. Traditionally, female participants have been overrepresented in surveys and interviews, suggested to be due to the gender differences in communication [25]. Surveys require a willingness to disclose some personal information and often having to express more socioemotional behaviors. These are traits that are historically characterized by females and may therefore contribute to their greater participation in survey research [25]. Moreover, when engaging on the internet, female users are more likely to communicate and exchange information, whereas male users prefer to information seek [26].

The advantage of using Facebook’s paid advertisement campaigns is that it can be set to target a specific audience, and set a daily cost limit. This is especially useful for researchers who are working within limited funding arrangements. Minimizing research costs and maximizing recruitment opportunities can be achieved with the use of social media for population health research. Social media recruitment desirability has also increased during the COVID-19 pandemic [27,28], with traditional methods unable to be used to recruit participants due to the public health measures used to combat the transmission of COVID-19.

Compared with the paid advertisement, the no-cost Facebook method of recruitment was time intensive, by virtue of having to contact administrators for permission to join groups and the ongoing posts and reposts to the group pages to ensure continued visibility. However, it can be said that traditional methods of participant recruitment such as mailed surveys are often more labor intensive and expensive [29]. A number of studies have been conducted comparing social media recruitment and traditional methods, suggesting that social media is more effective for cost and time [16,17,30]. Indeed, social media recruitment through both the paid and no-cost options, as demonstrated in this study, represent a cost-effective method of recruitment into a population health survey.

Surprisingly, in week 7, a total of 198 responses were received; this coincided with a long weekend in 3 Australian States (New South Wales, Queensland, and South Australia) and one territory (Australian Capital Territory) and may have increased the response rates in this week. This finding suggests that targeting social media recruitment over weekends and when people have spare time, particularly during the COVID-19 pandemic when people may have been in lockdown over the long weekend, may provide a good opportunity for recruitment.

### Limitations

Although this study used robust methods, there are some limitations that need to be acknowledged. First, there is potential for bias due to exposure to the advertisement being associated with time spent on Facebook (and therefore not the same for each user), especially with the community noticeboard groups where visibility of the post depended on when potential respondents were on Facebook.

Second, the feasibility of Facebook as a recruitment tool can be impacted by Facebook’s automated advertising algorithms and metrics. Facebook sets advertising algorithms to determine the most appropriate advertisements to show to a specific audience. However, this is also impacted by Facebook as a business wanting to provide the user with a good experience. The metrics used by Facebook can be difficult to comprehend, which in turn can be challenging for researchers, particularly when they are not familiar with interpreting the metrics or following previously published social media recruitment protocols.

Third, only one online survey link was established for this study, which meant that being able to track respondents from each recruitment option was impossible. Future research employing both no-cost and paid options should use two separate links to enable a more robust comparison of the two options.

Despite male participants engaging with the Facebook advertisement campaigns more than women, they are underrepresented in this study. Approaches to increase male participation in online surveys needs to be explored.

Finally, further qualitative studies need to be conducted to understand why individuals choose or decline to participant in research advertised through social media.

### Conclusions

Recruitment through social media, specifically Facebook, allowed for a cost-effective and efficient method for recruiting a national sample of participants for a web-based survey about the relationship between well-being and the social determinants of health during the COVID-19 pandemic. The diversity of participants recruited in this study, in terms of socioeconomic status, remoteness, educational attainment, and age, promotes and confirms the feasibility of social media to recruit hard-to-reach population groups as well as a diverse sample of the national population. The benefits of using Facebook should be considered by population health researchers when implementing health research in the future.

## Abbreviations

ASB: Australian Bureau of Statistics
IRSAD: Index of Relative Socio-Economic Advantage and Disadvantage
